# Electronic Pupillometry Supporting the Diagnosis of Adie’s Tonic Pupil Following Pterygium Surgery

**DOI:** 10.7759/cureus.109418

**Published:** 2026-05-22

**Authors:** Peter J Tweedie, Amitouj S Sidhu, Lawrence W Hurst, Ian C Francis

**Affiliations:** 1 Faculty of Medicine, University of Western Australia, Perth, AUS; 2 Department of Ophthalmology, Westmead Hospital, Sydney, AUS; 3 Ophthalmology, Queensland Eye Institute, The University of Queensland, and The Australian Pterygium Centre, Brisbane, AUS; 4 Department of Ophthalmology, Prince of Wales Hospital, Sydney, AUS

**Keywords:** adie’s tonic pupil, automated pupillometer, holmes adie syndrome, pterygium excision, pterygium surgery

## Abstract

This article reports the case of a 40-year-old man who underwent right primary pterygium surgery, resulting in the successful removal of the pterygium and a good cosmetic outcome. However, one month after surgery, the patient noticed that his right pupil was enlarged. He was referred for a neuro-ophthalmologic examination, and a diagnosis of Holmes-Adie syndrome was made. The pupil abnormality was considered unlikely to be iatrogenic from the surgery. Both of the patient’s pupils demonstrated light-near dissociation due to bilateral Holmes-Adie syndrome, indicating a pre-existing, previously undiagnosed tonic pupil in the left eye. The pupillary findings were confirmed using the handheld NPi-200 electronic pupillometer.

## Introduction

A pterygium is a fleshy overgrowth of the conjunctiva and associated fibrous tissue onto the cornea, usually caused by ultraviolet light exposure [1]. PERFECT pterygium surgery was developed by Professor Lawrence W. Hirst from Queensland, Australia, after he observed that the recurrence rate following pterygium surgery at his local Brisbane hospital was 46%, even with adjunctive radiotherapy [2]. PERFECT is an acronym for Pterygium Extended Removal Followed By Extended Conjunctival Transplant. PERFECT surgery demonstrates a virtually zero recurrence rate with excellent cosmetic outcomes. Reported complications were rare: one patient developed exotropia that required no treatment, and another patient lost four lines of best-corrected visual acuity due to a corneal ulcer following surgery [3].

## Case presentation

**Figure 1:**
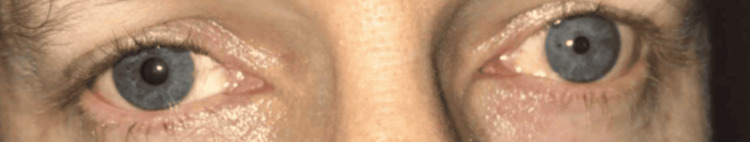
The patient's eyes one month after PERFECT surgery, demonstrating right pupillary mydriasis PERFECT: Pterygium Extended Removal Followed By Extended Conjunctival Transplant

A 40-year-old man underwent PERFECT surgery for a right nasal primary pterygium. He had no significant family history of systemic or ophthalmologic diseases, no allergies, no history of ocular or head trauma, and was in good general health. He had previously undergone successful and uncomplicated left pterygium surgery 20 years earlier at another center. The right pterygium was successfully excised using PERFECT, with a good cosmetic outcome. Visual acuity in both eyes was logMAR 0 (Snellen 6/6) before and after surgery. One month later, the patient noticed enlargement of his right pupil and was concerned about the resulting anisocoria (Figure 1). Review of old photographs demonstrated previously isocoric pupils. The surgeon was unable to explain the findings and referred the patient to a neuro-ophthalmologist.

Neuro-ophthalmologic examination disclosed no tropia or diplopia, and ocular rotations were normal. Visual fields by confrontation, slit-lamp examination, and fundi were unremarkable, and all cranial nerves were intact except for the pupils. Both pupils were relatively unreactive to light, but constricted well with accommodation, demonstrating bilateral light-near dissociation. Vermiform movements of the iris were noted in the right eye on slit-lamp examination. Tonic re-dilation of the pupil was observed when the patient looked at a distant target. Further neuro-ophthalmologic examination demonstrated normal lower limb sweating, but bilateral absence of knee and ankle reflexes.

The patient’s pupil diameters were measured using the NPi-200® electronic pupillometer, and again 30 minutes after instillation of dilute pilocarpine (0.1%) to test for denervation hypersensitivity. A pupillometer objectively measures pupil size and is more accurate than manual measurement [4]. Initially, his pupils measured 4.69 mm OD and 3.56 mm OS. Thirty minutes later, both pupils constricted, with the smaller left pupil constricting more than the larger right pupil, to 4.09 mm OD and 2.48 mm OS, demonstrating denervation hypersensitivity both objectively and accurately. This represented a percentage reduction in pupil diameter of 12.8% OD and 30.3% OS, corresponding to a percentage reduction in pupil area of 13.1% OD and 51.5% OS (Table 1).

**Table 1:** Pupil sizes before and after topical administration of dilute pilocarpine, as measured by electronic pupillometry

Pupil diameter	Right eye	Left eye
Before pilocarpine	4.69 mm	3.56 mm
30 minutes after pilocarpine	4.09 mm	2.48 mm

Thus, based on the findings of light-near dissociation, denervation hypersensitivity, and absent lower limb deep tendon reflexes, a diagnosis of bilateral Holmes-Adie syndrome was made by the primary neuro-ophthalmologist, with a recent-onset right Adie’s pupil and a longstanding left Adie’s pupil. The patient opted for a second opinion from another neuro-ophthalmologist, who ordered an MRI of the brain and orbits, which was normal.

## Discussion

Holmes-Adie syndrome is an autonomic parasympathetic disorder localised to the ciliary ganglion [4]. It comprises Adie’s tonic pupil and is associated with deep tendon areflexia in the lower limbs, symptomatic postural hypotension, and preserved lower limb sweating. Over time, a longstanding Adie’s tonic pupil can decrease in size due to chronic denervation, a phenomenon referred to as a “little old Adie”. Note of normal sweating is important, as a differential diagnosis is Ross syndrome, which involves the same autonomic signs as Holmes-Adie syndrome, with the addition of anhidrosis [5].

In our patient, the diagnosis of Holmes-Adie syndrome with bilateral pupillary involvement, including a new right Adie’s pupil following PERFECT pterygium surgery, was determined to be unrelated to the surgery itself. Direct patient questioning indicated that he had no recollection of previously having a large left pupil at the time of onset of his left Adie’s pupil, which has now evolved into a “little old Adie.” Differential diagnoses of light-near dissociation include third cranial nerve palsy, dorsal midbrain syndrome, relative afferent pupillary defect, most optic neuropathies, aberrant regeneration of the third nerve, iris trauma (including laser photocoagulation and blunt trauma), chronic alcoholism, Riley-Day syndrome, Argyll Robertson pupil, Wernicke’s encephalopathy, herpes zoster ophthalmicus, and myotonic dystrophy.

Holmes-Adie syndrome is usually seen in females, with a 2:1 female-to-male ratio, most commonly in their third and fourth decades [6]. There have been a few reports in the literature of coincidental or iatrogenic Adie’s tonic pupil following surgery. Sirikurama et al. in 1990 [7] reported a case of Adie’s pupil following Le Fort I maxillary osteotomy in a 24-year-old female, but they were unable to determine whether it was a complication of the surgery or merely coincidental. An iatrogenic tonic pupil was reported by Ebrahim et al. in 2009 [8] following retinal detachment repair, attributed to injury to the short ciliary nerves by endolaser treatment. Akahane et al. also reported an iatrogenic case of bilateral Adie’s pupils following panretinal photocoagulation for uveitis, due to laser damage around the ciliary ganglion [9].

## Conclusions

This case appears to be unique in providing a comprehensive, yet coincidental, presentation of Holmes-Adie syndrome following the extensive conjunctival dissection of PERFECT pterygium surgery. It is noteworthy that a pre-existing Adie’s tonic pupil in the contralateral eye was present but previously undiagnosed. Accordingly, the patient’s acute presentation of monocular mydriasis was coincidental, and the temporal association with surgery does not imply iatrogenic causation.
